# Supplementary material for: A Humanized Yeast Phenomic Model of Deoxycytidine Kinase to Predict Genetic Buffering of Nucleoside Analog Cytotoxicity
Source: Genes (Basel). 2019 Sep 30;10(10):770. doi: 10.3390/genes10100770 (PMC6826991; doi:10.3390/genes10100770)
Supplement: Supplementary file 1 [file genes-10-00770-s001.zip › Additional_Files/Additional_File7_GOTermHeatmaps.zip!/Additional_File7_GOTermHeatmaps/regulation_of_filamentous_growth.pdf]

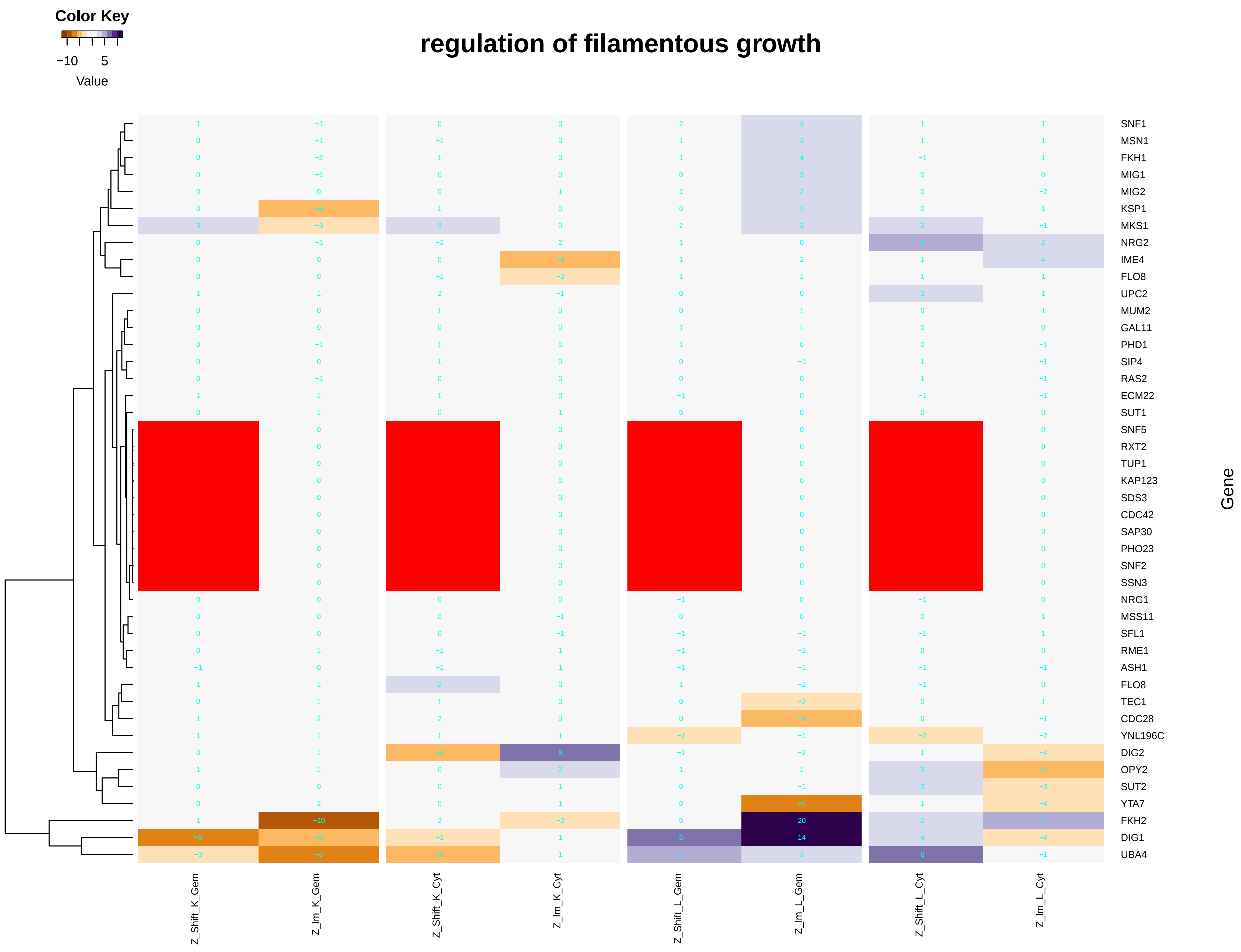
Color Key
−10
5
Value
regulation of filamentous growth
SNF1
MSN1
FKH1
MIG1
MIG2
KSP1
MKS1
NRG2
IME4
FLO8
UPC2
MUM2
GAL11
PHD1
SIP4
RAS2
ECM22
SUT1
SNF5
RXT2
TUP1
KAP123
SDS3
CDC42
SAP30
PHO23
SNF2
SSN3
NRG1
MSS11
SFL1
RME1
ASH1
FLO8
TEC1
CDC28
YNL196C
DIG2
OPY2
SUT2
YTA7
FKH2
DIG1
UBA4
Gene
Z_Shift_K_Gem
Z_Im_K_Gem
Z_Shift_K_Cyt
Z_Im_K_Cyt
Z_Shift_L_Gem
Z_Im_L_Gem
Z_Shift_L_Cyt
Z_Im_L_Cyt

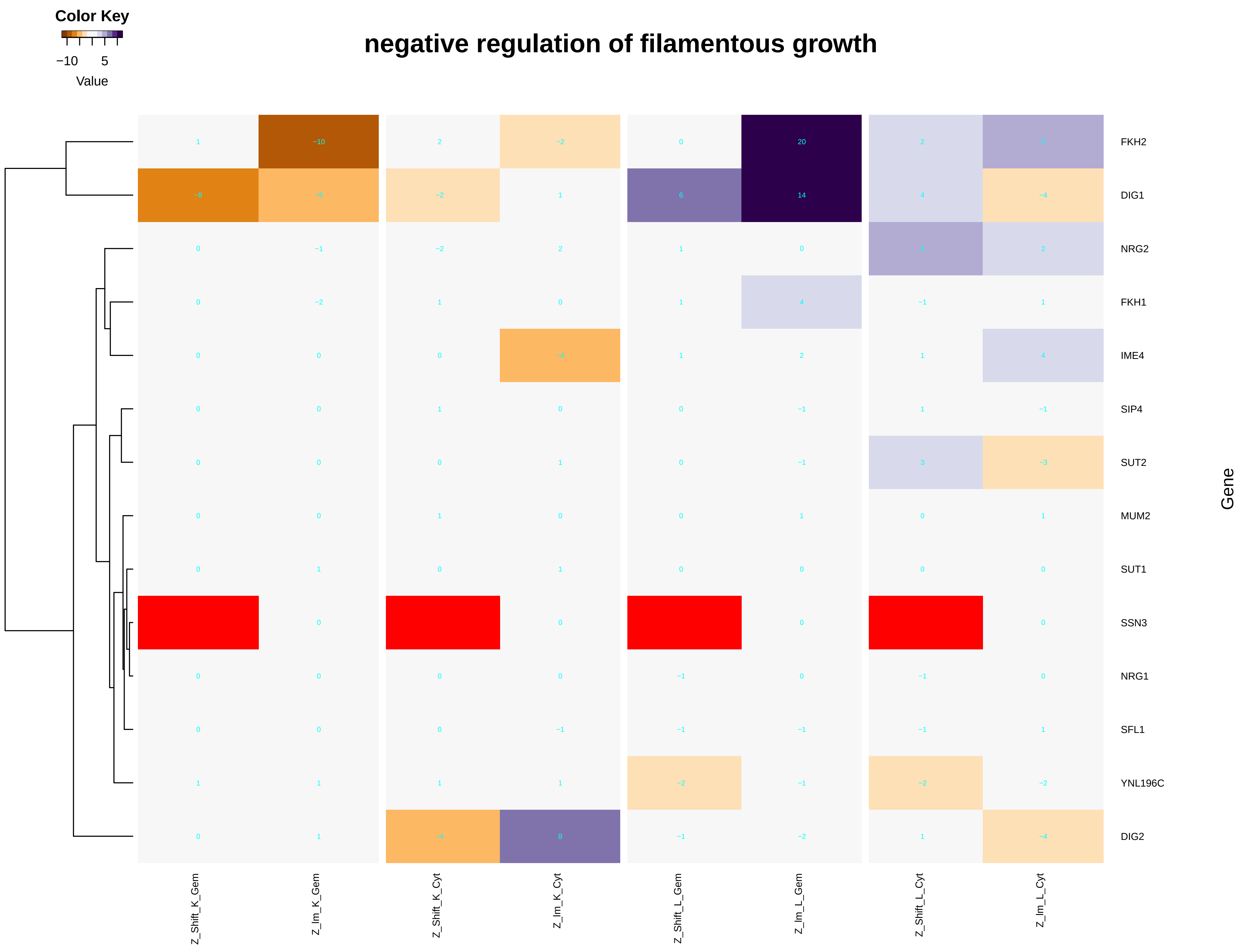
Color Key
−10 5
Value
negative regulation of filamentous growth
FKH2
DIG1
NRG2
FKH1
IME4
SIP4
SUT2
MUM2
SUT1
SSN3
NRG1
SFL1
YNL196C
DIG2
Gene
Z_Shift_K_Gem
Z_Im_K_Gem
Z_Shift_K_Cyt
Z_Im_K_Cyt
Z_Shift_L_Gem
Z_Im_L_Gem
Z_Shift_L_Cyt
Z_Im_L_Cyt

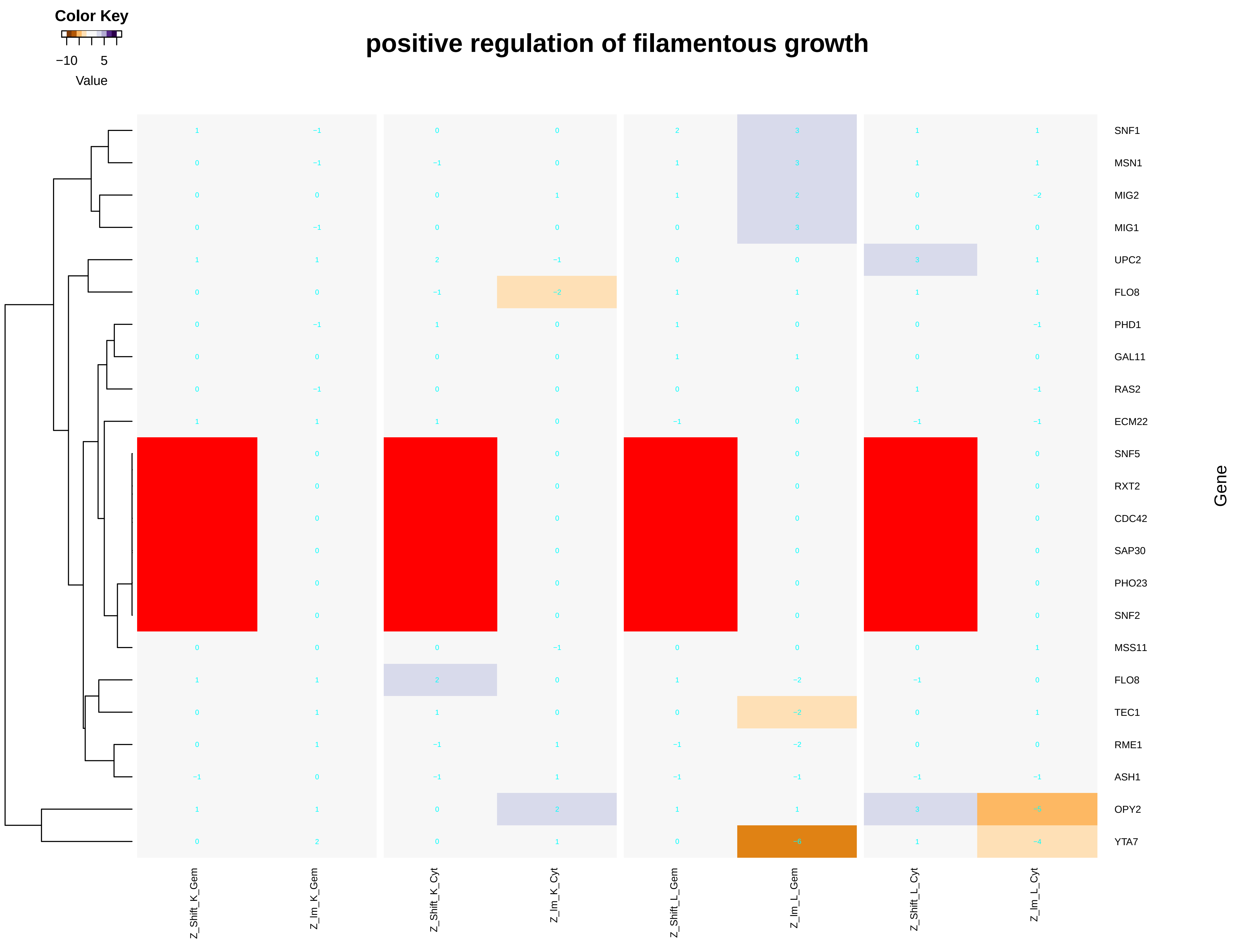

Color Key
−10
5
Value
positive regulation of filamentous growth
SNF1
MSN1
MIG2
MIG1
UPC2
FLO8
PHD1
GAL11
RAS2
ECM22
SNF5
RXT2
CDC42
SAP30
PHO23
SNF2
MSS11
FLO8
TEC1
RME1
ASH1
OPY2
YTA7
Gene
Z_Shift_K_Gem
Z_Im_K_Gem
Z_Shift_K_Cyt
Z_Im_K_Cyt
Z_Shift_L_Gem
Z_Im_L_Gem
Z_Shift_L_Cyt
Z_Im_L_Cyt

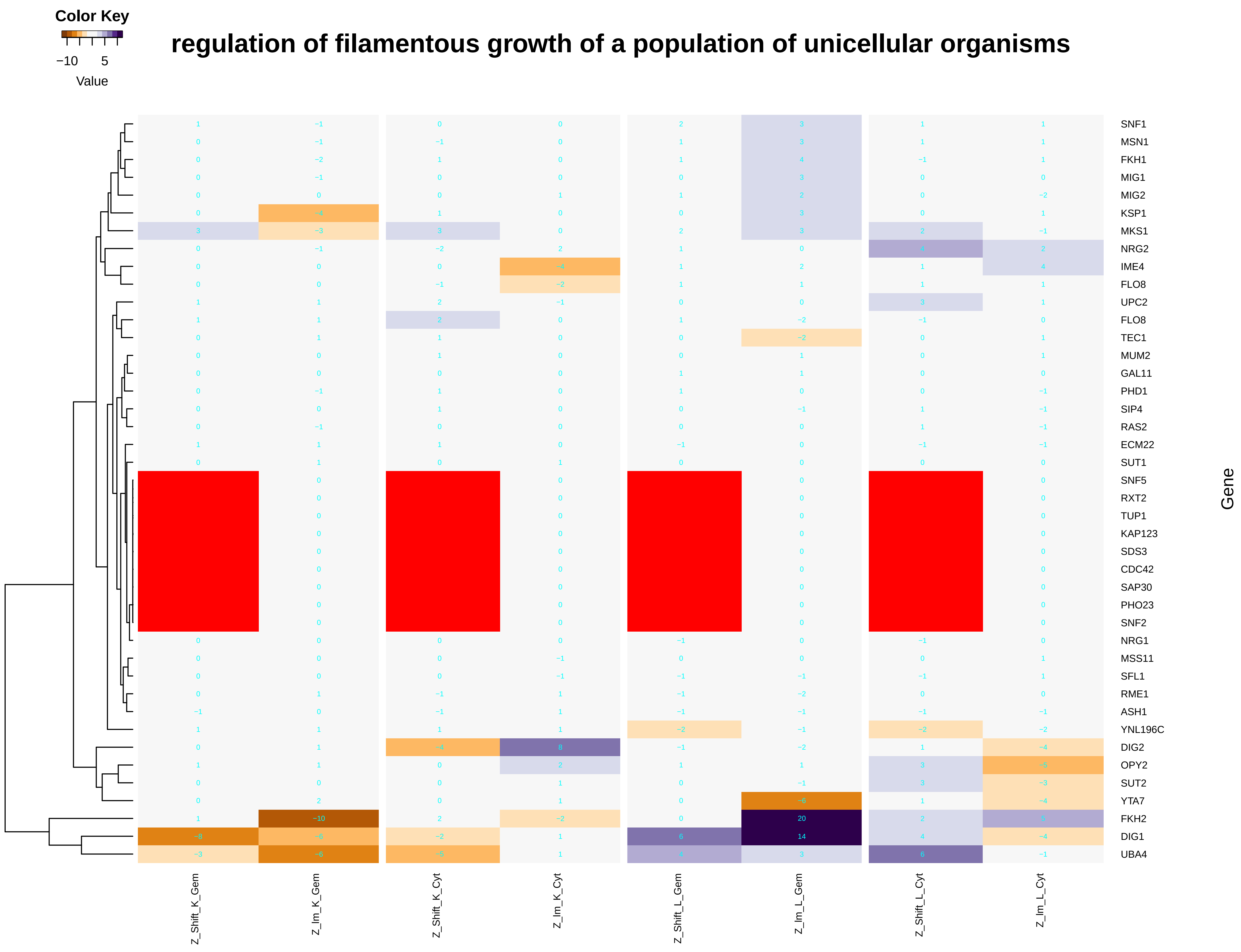
Color Key
−10 5
Value
regulation of filamentous growth of a population of unicellular organisms
SNF1
MSN1
FKH1
MIG1
MIG2
KSP1
MKS1
NRG2
IME4
FLO8
UPC2
FLO8
TEC1
MUM2
GAL11
PHD1
SIP4
RAS2
ECM22
SUT1
SNF5
RXT2
TUP1
KAP123
SDS3
CDC42
SAP30
PHO23
SNF2
NRG1
MSS11
SFL1
RME1
ASH1
YNL196C
DIG2
OPY2
SUT2
YTA7
FKH2
DIG1
UBA4
Gene
Z_Shift_K_Gem
Z_Im_K_Gem
Z_Shift_K_Cyt
Z_Im_K_Cyt
Z_Shift_L_Gem
Z_Im_L_Gem
Z_Shift_L_Cyt
Z_Im_L_Cyt

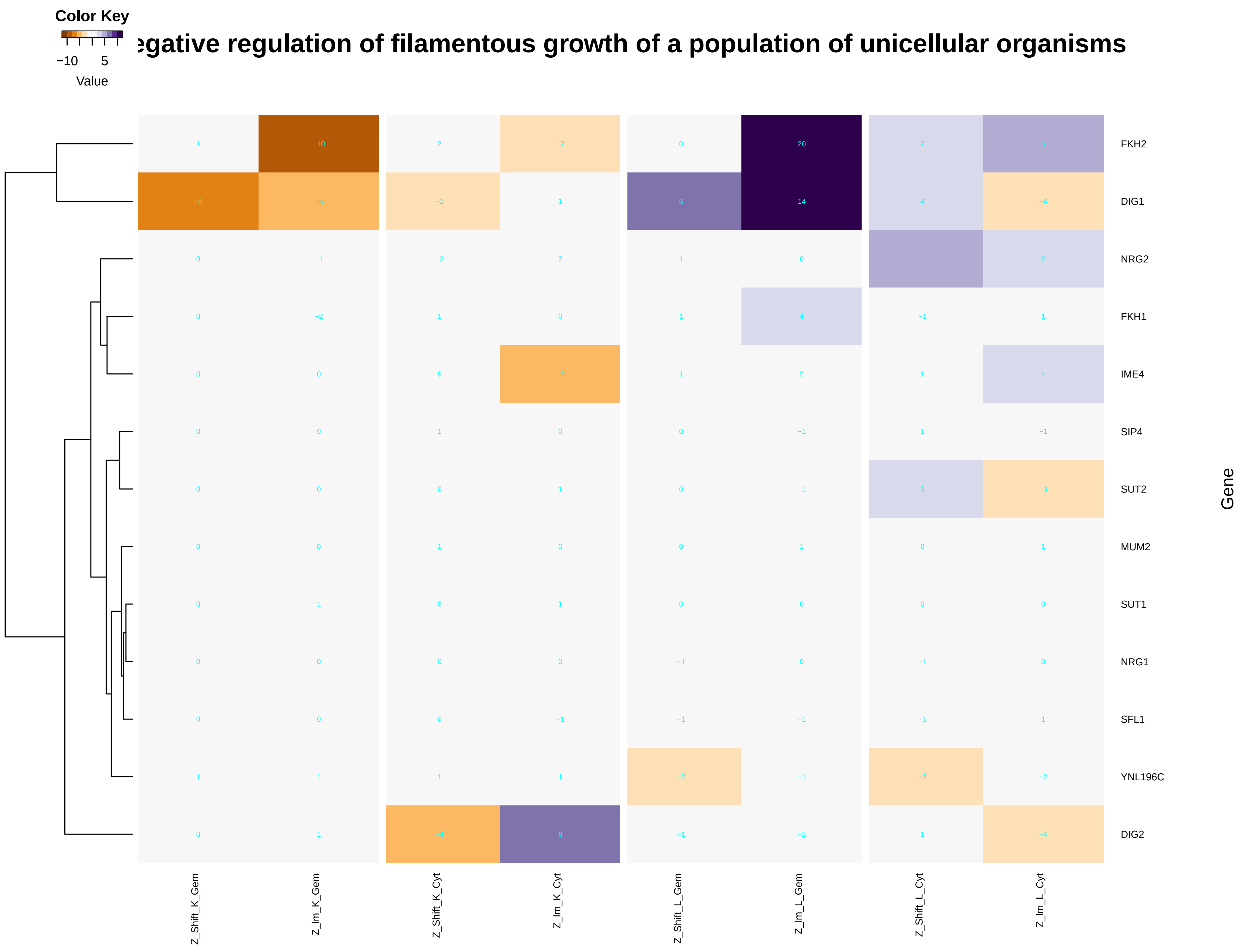

Color Key
−10
5
Value
egative regulation of filamentous growth of a population of unicellular organisms
FKH2
DIG1
NRG2
FKH1
IME4
SIP4
SUT2
MUM2
SUT1
NRG1
SFL1
YNL196C
DIG2
Gene
Z_Shift_K_Gem
Z_Im_K_Gem
Z_Shift_K_Cyt
Z_Im_K_Cyt
Z_Shift_L_Gem
Z_Im_L_Gem
Z_Shift_L_Cyt
Z_Im_L_Cyt

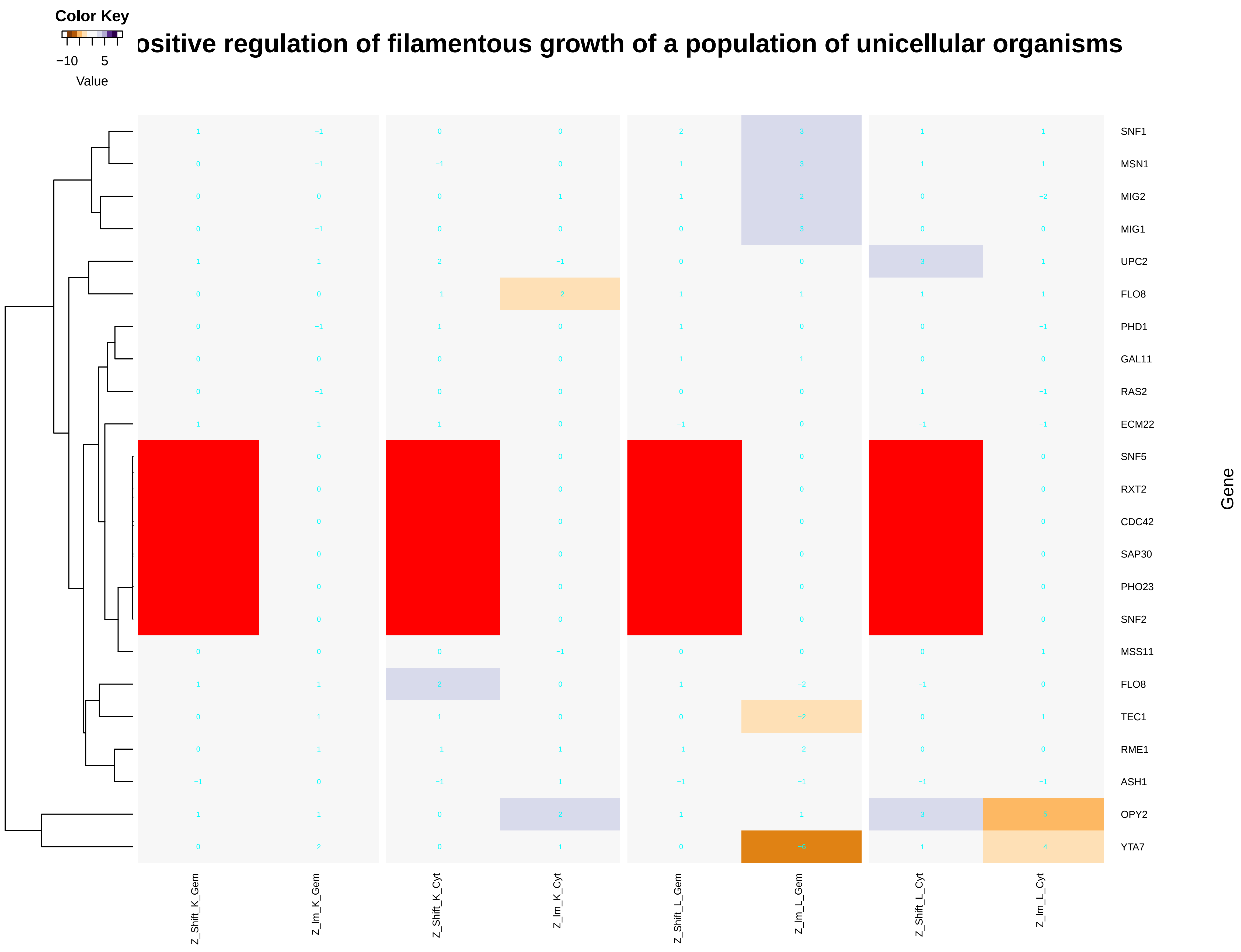

ositive regulation of filamentous growth of a population of unicellular organisms
Color Key
−10
5
Value
SNF1
MSN1
MIG2
MIG1
UPC2
FLO8
PHD1
GAL11
RAS2
ECM22
SNF5
RXT2
CDC42
SAP30
PHO23
SNF2
MSS11
FLO8
TEC1
RME1
ASH1
OPY2
YTA7
Gene
Z_Shift_K_Gem
Z_lm_K_Gem
Z_Shift_K_Cyt
Z_lm_K_Cyt
Z_Shift_L_Gem
Z_lm_L_Gem
Z_Shift_L_Cyt
Z_lm_L_Cyt

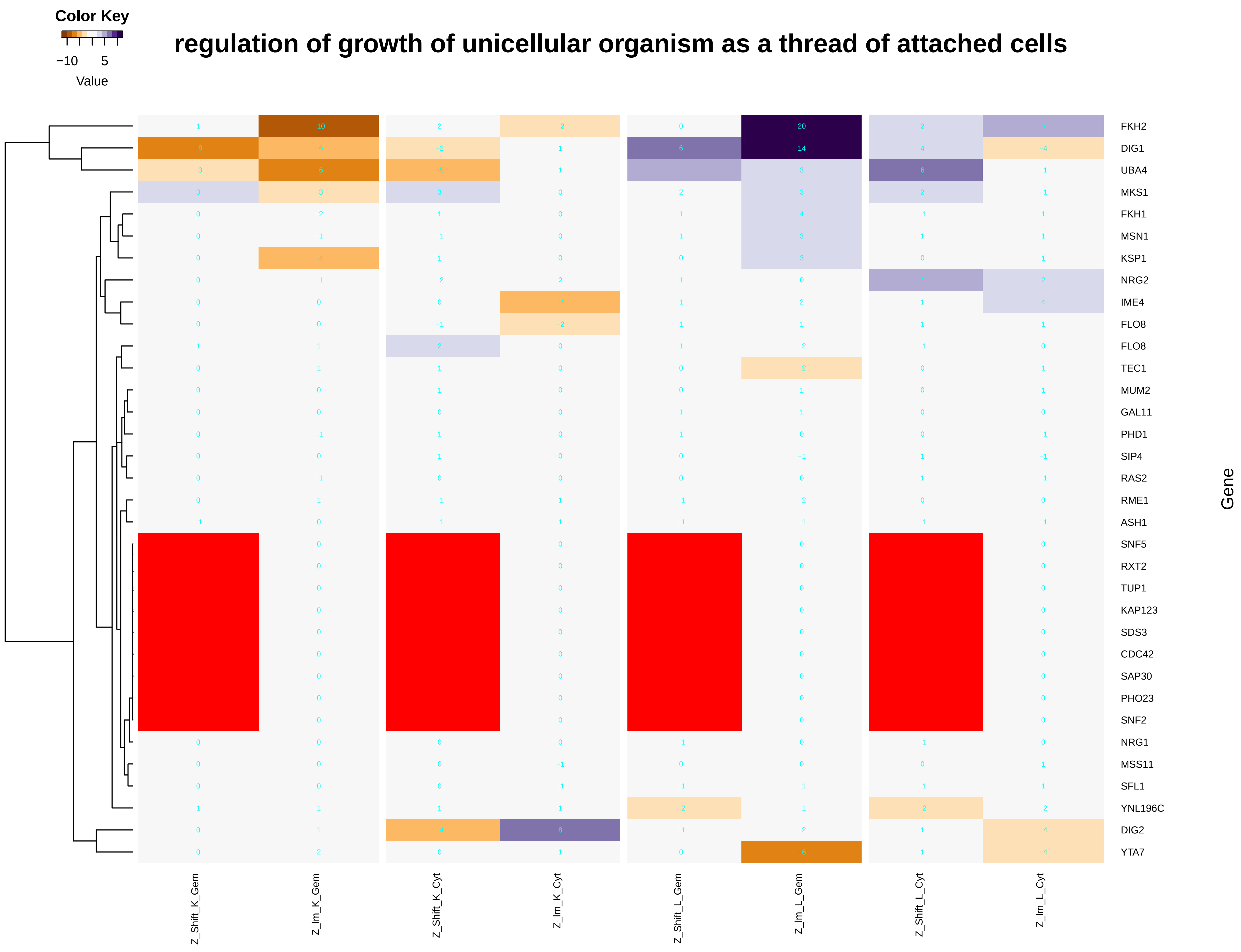
Color Key
−10
5
Value
regulation of growth of unicellular organism as a thread of attached cells
FKH2
DIG1
UBA4
MKS1
FKH1
MSN1
KSP1
NRG2
IME4
FLO8
FLO8
TEC1
MUM2
GAL11
PHD1
SIP4
RAS2
RME1
ASH1
SNF5
RXT2
TUP1
KAP123
SDS3
CDC42
SAP30
PHO23
SNF2
NRG1
MSS11
SFL1
YNL196C
DIG2
YTA7
Gene
Z_Shift_K_Gem
Z_Im_K_Gem
Z_Shift_K_Cyt
Z_Im_K_Cyt
Z_Shift_L_Gem
Z_Im_L_Gem
Z_Shift_L_Cyt
Z_Im_L_Cyt

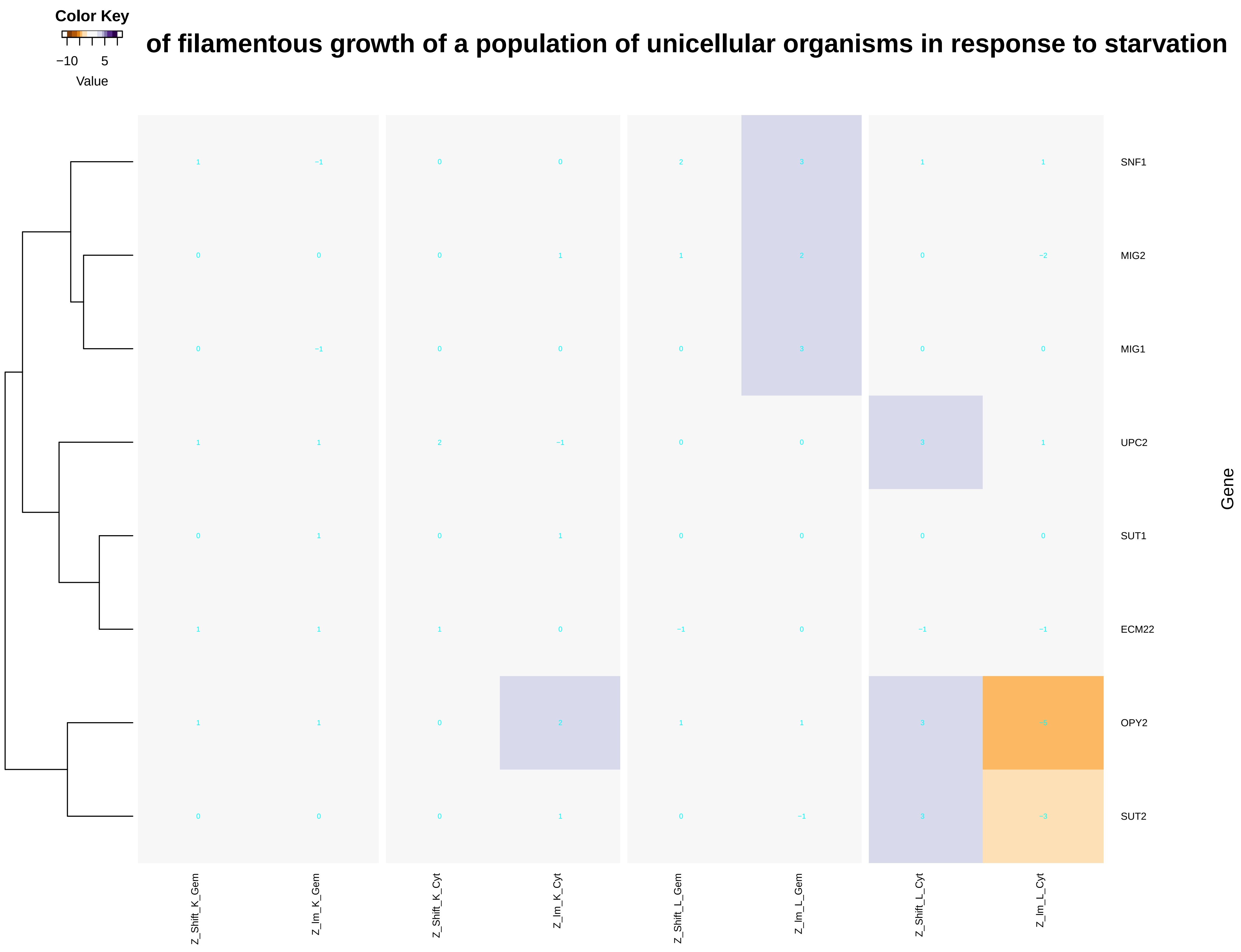
Color Key
−10
5
Value
of filamentous growth of a population of unicellular organisms in response to starvation
SNF1
MIG2
MIG1
UPC2
SUT1
ECM22
OPY2
SUT2
Gene
Z_Shift_K_Gem
Z_Im_K_Gem
Z_Shift_K_Cyt
Z_Im_K_Cyt
Z_Shift_L_Gem
Z_Im_L_Gem
Z_Shift_L_Cyt
Z_Im_L_Cyt

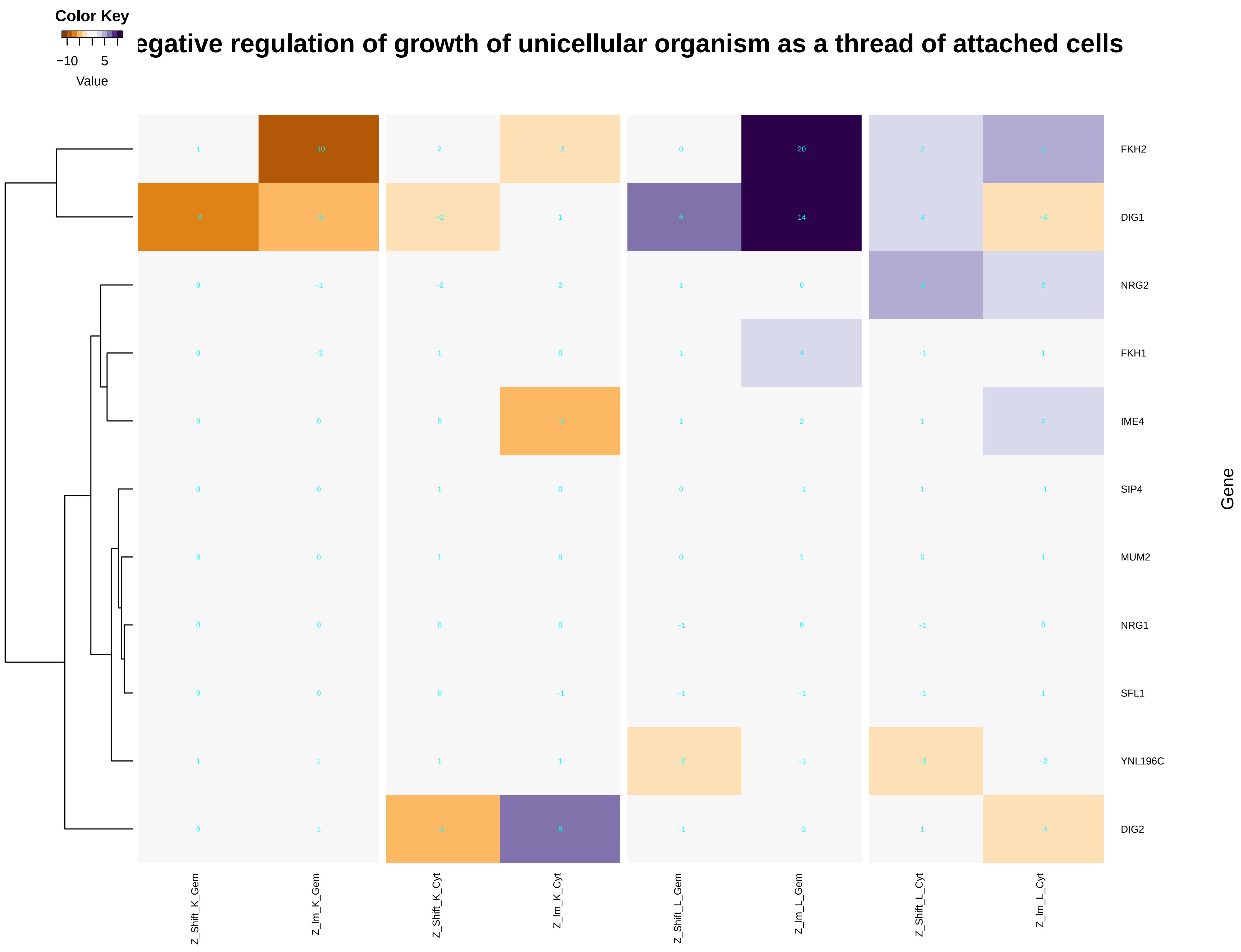
Color Key
−10
5
Value
egative regulation of growth of unicellular organism as a thread of attached cells
FKH2
DIG1
NRG2
FKH1
IME4
SIP4
MUM2
NRG1
SFL1
YNL196C
DIG2
Gene
Z_Shift_K_Gem
Z_Im_K_Gem
Z_Shift_K_Cyt
Z_Im_K_Cyt
Z_Shift_L_Gem
Z_Im_L_Gem
Z_Shift_L_Cyt
Z_Im_L_Cyt

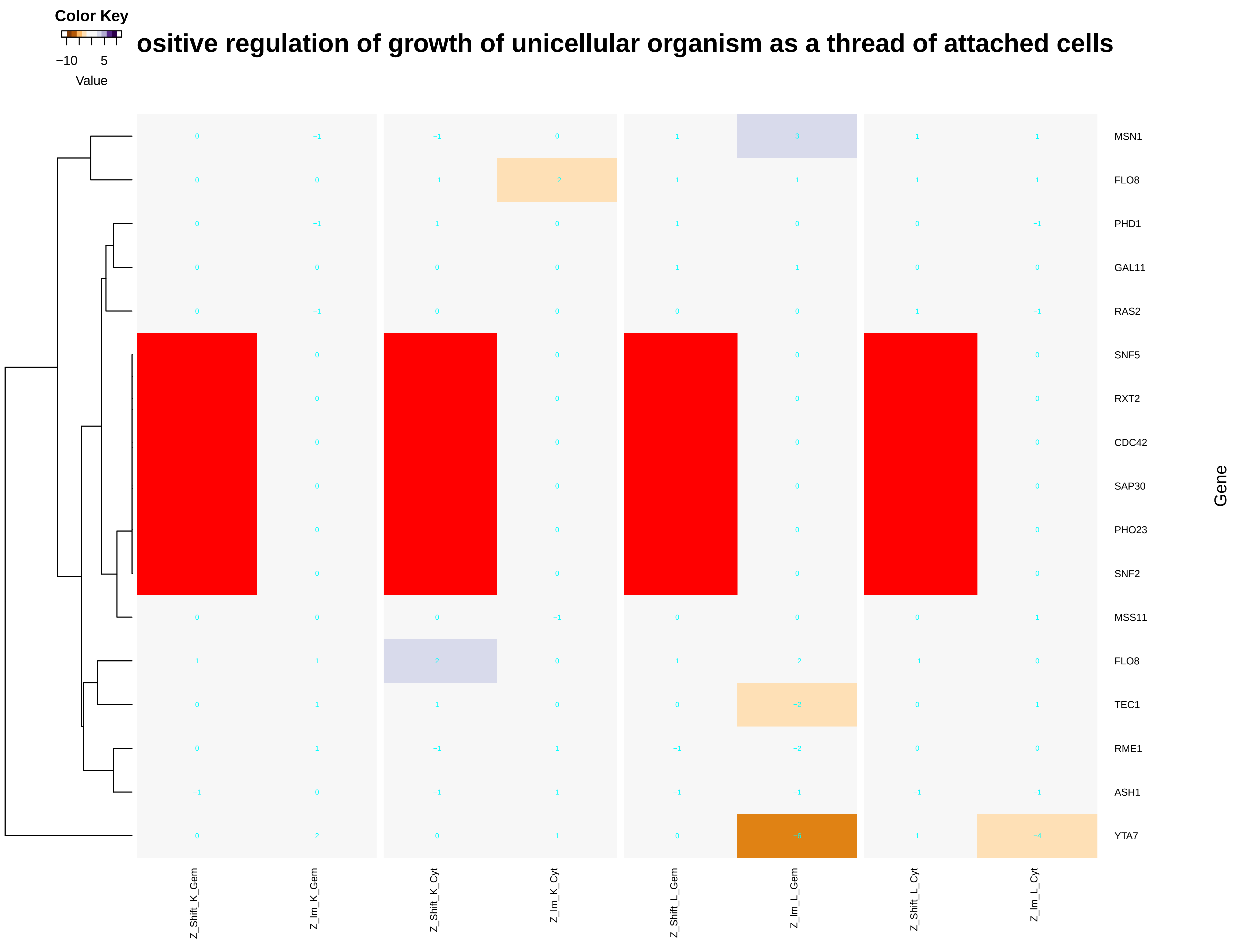

Color Key
−10 5
Value
ositive regulation of growth of unicellular organism as a thread of attached cells
MSN1
FLO8
PHD1
GAL11
RAS2
SNF5
RXT2
CDC42
SAP30
PHO23
SNF2
MSS11
FLO8
TEC1
RME1
ASH1
YTA7
Gene
Z_Shift_K_Gem
Z_Im_K_Gem
Z_Shift_K_Cyt
Z_Im_K_Cyt
Z_Shift_L_Gem
Z_Im_L_Gem
Z_Shift_L_Cyt
Z_Im_L_Cyt

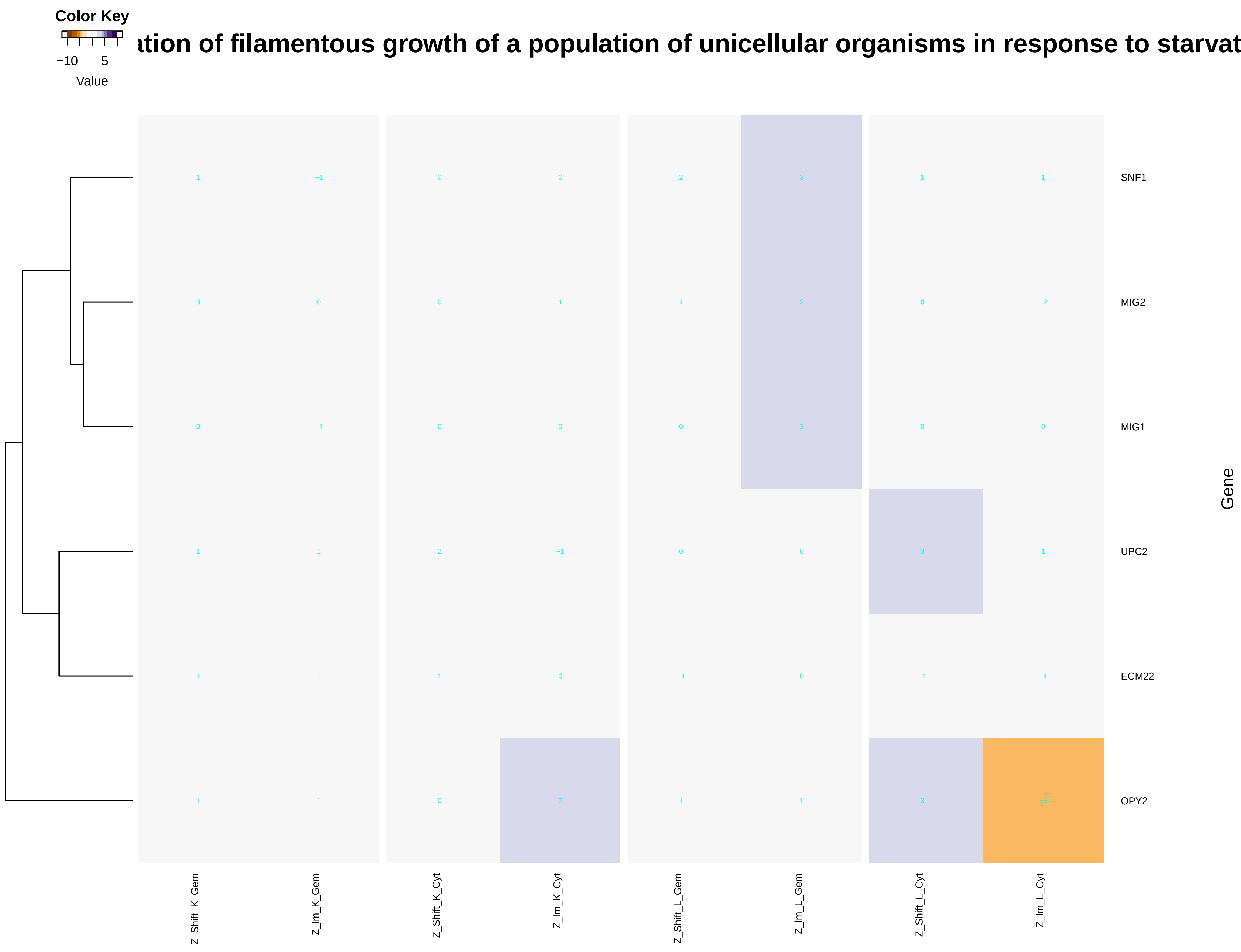
Color Key
−10 5
Value
ation of filamentous growth of a population of unicellular organisms in response to starvat
SNF1
MIG2
MIG1
UPC2
ECM22
OPY2
Gene
Z_Shift_K_Gem
Z_Im_K_Gem
Z_Shift_K_Cyt
Z_Im_K_Cyt
Z_Shift_L_Gem
Z_Im_L_Gem
Z_Shift_L_Cyt
Z_Im_L_Cyt

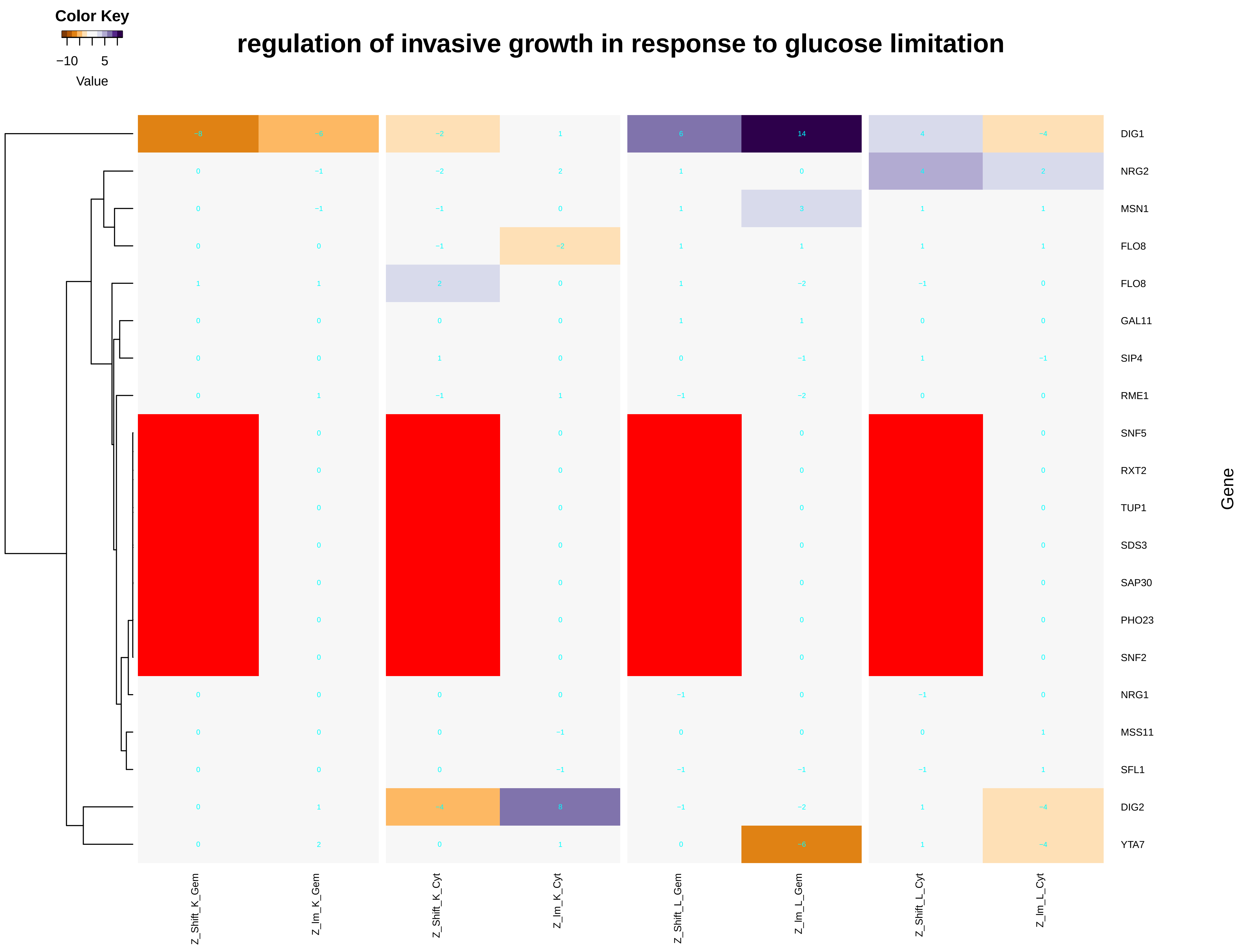

Color Key
−10
5
Value
regulation of invasive growth in response to glucose limitation
DIG1
NRG2
MSN1
FLO8
FLO8
GAL11
SIP4
RME1
SNF5
RXT2
TUP1
SDS3
SAP30
PHO23
SNF2
NRG1
MSS11
SFL1
DIG2
YTA7
Gene
Z_Shift_K_Gem
Z_Im_K_Gem
Z_Shift_K_Cyt
Z_Im_K_Cyt
Z_Shift_L_Gem
Z_Im_L_Gem
Z_Shift_L_Cyt
Z_Im_L_Cyt

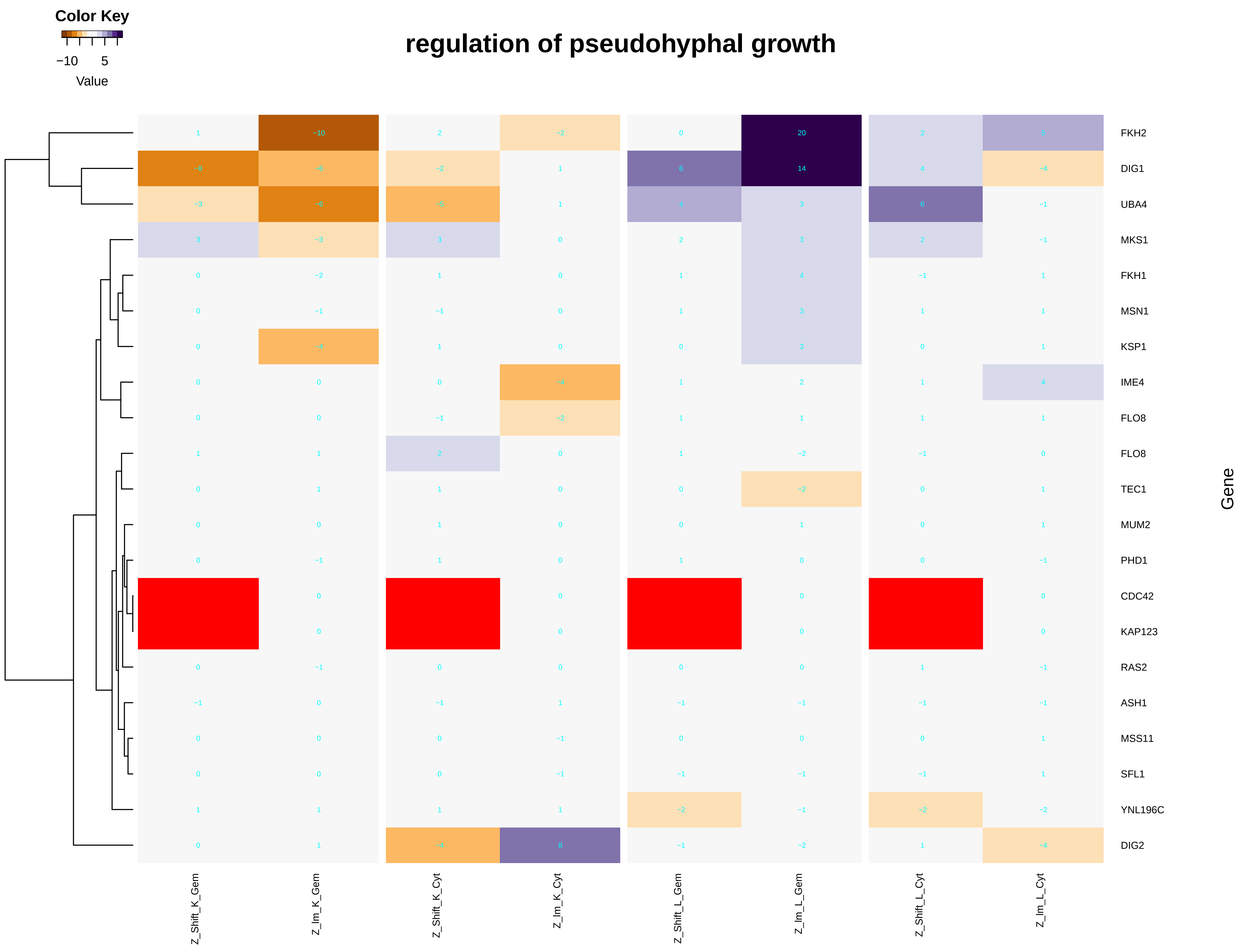
Color Key
−10
5
Value
regulation of pseudohyphal growth
FKH2
DIG1
UBA4
MKS1
FKH1
MSN1
KSP1
IME4
FLO8
FLO8
TEC1
MUM2
PHD1
CDC42
KAP123
RAS2
ASH1
MSS11
SFL1
YNL196C
DIG2
Gene
Z_Shift_K_Gem
Z_Im_K_Gem
Z_Shift_K_Cyt
Z_Im_K_Cyt
Z_Shift_L_Gem
Z_Im_L_Gem
Z_Shift_L_Cyt
Z_Im_L_Cyt

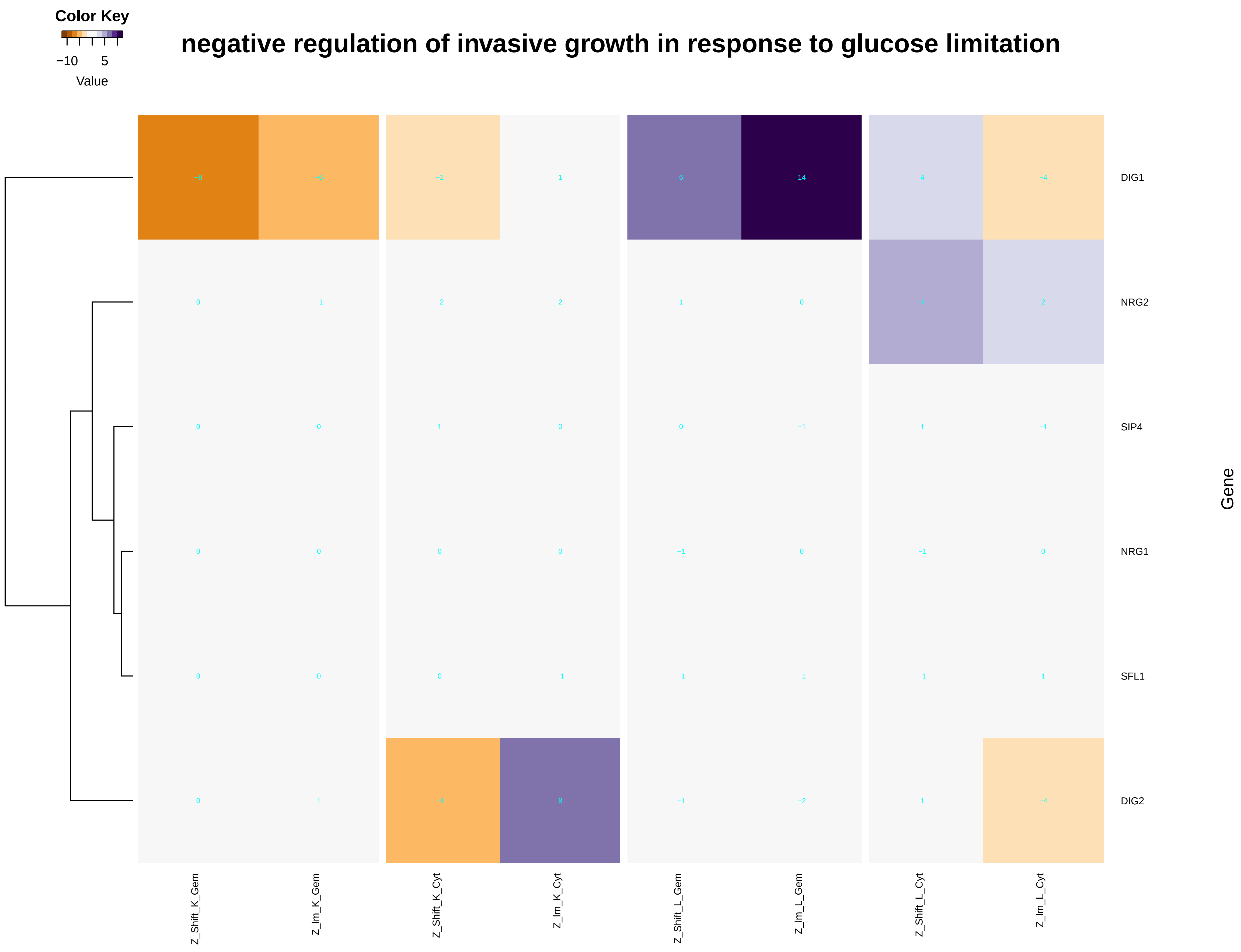

Color Key
−10
5
Value
negative regulation of invasive growth in response to glucose limitation
DIG1 −8 −6 −2 1 6 14 4 −4
NRG2 0 −1 −2 2 1 0 4 2
SIP4 0 0 1 0 0 −1 1 −1
NRG1 0 0 0 0 −1 0 −1 0
SFL1 0 0 0 −1 −1 −1 −1 1
DIG2 0 1 −4 8 −1 −2 1 −4
Gene
Z_Shift_K_Gem
Z_lm_K_Gem
Z_Shift_K_Cyt
Z_lm_K_Cyt
Z_Shift_L_Gem
Z_lm_L_Gem
Z_Shift_L_Cyt
Z_lm_L_Cyt

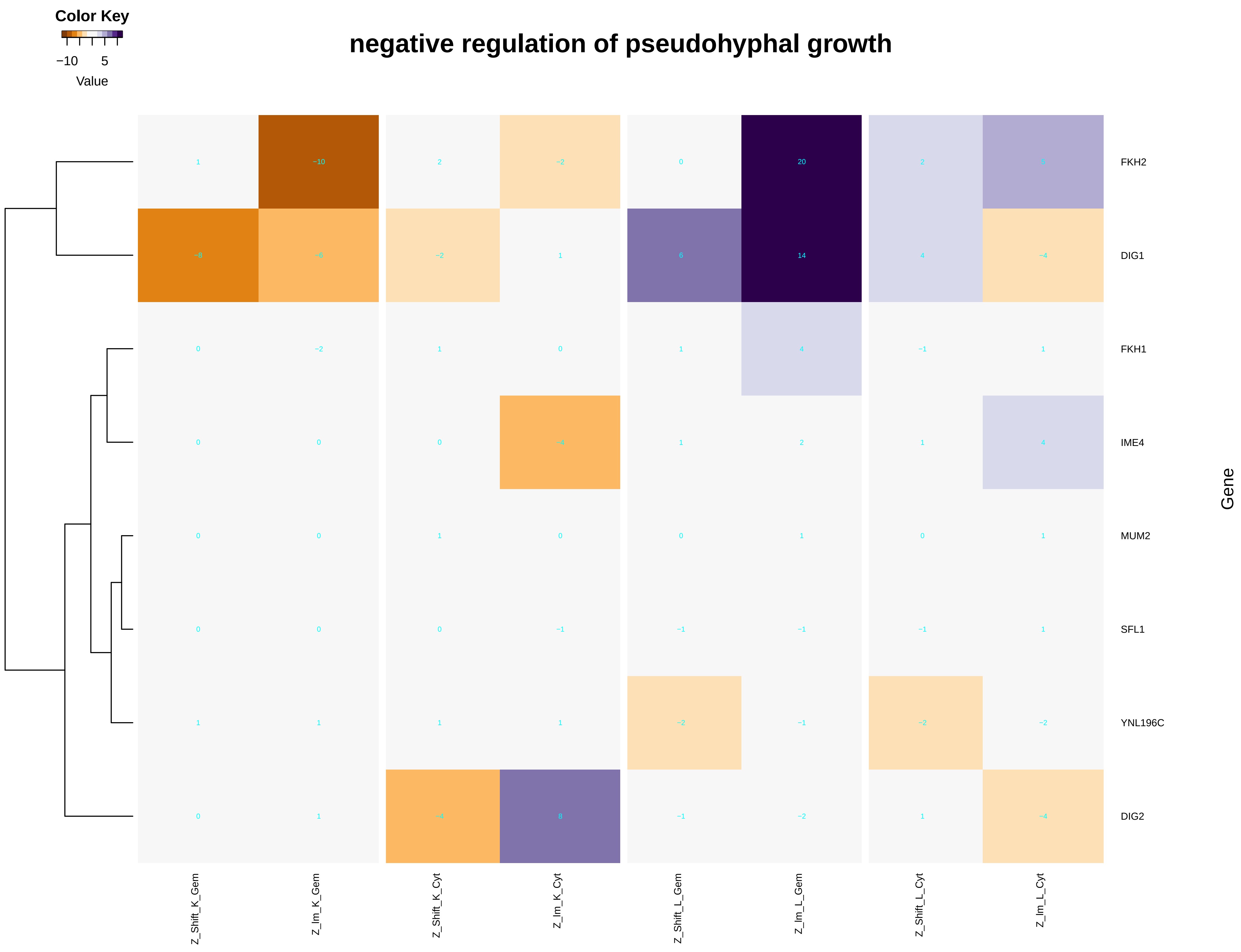

Color Key
−10
5
Value
negative regulation of pseudohyphal growth
FKH2
DIG1
FKH1
IME4
MUM2
SFL1
YNL196C
DIG2
Gene
Z_Shift_K_Gem
Z_Im_K_Gem
Z_Shift_K_Cyt
Z_Im_K_Cyt
Z_Shift_L_Gem
Z_Im_L_Gem
Z_Shift_L_Cyt
Z_Im_L_Cyt

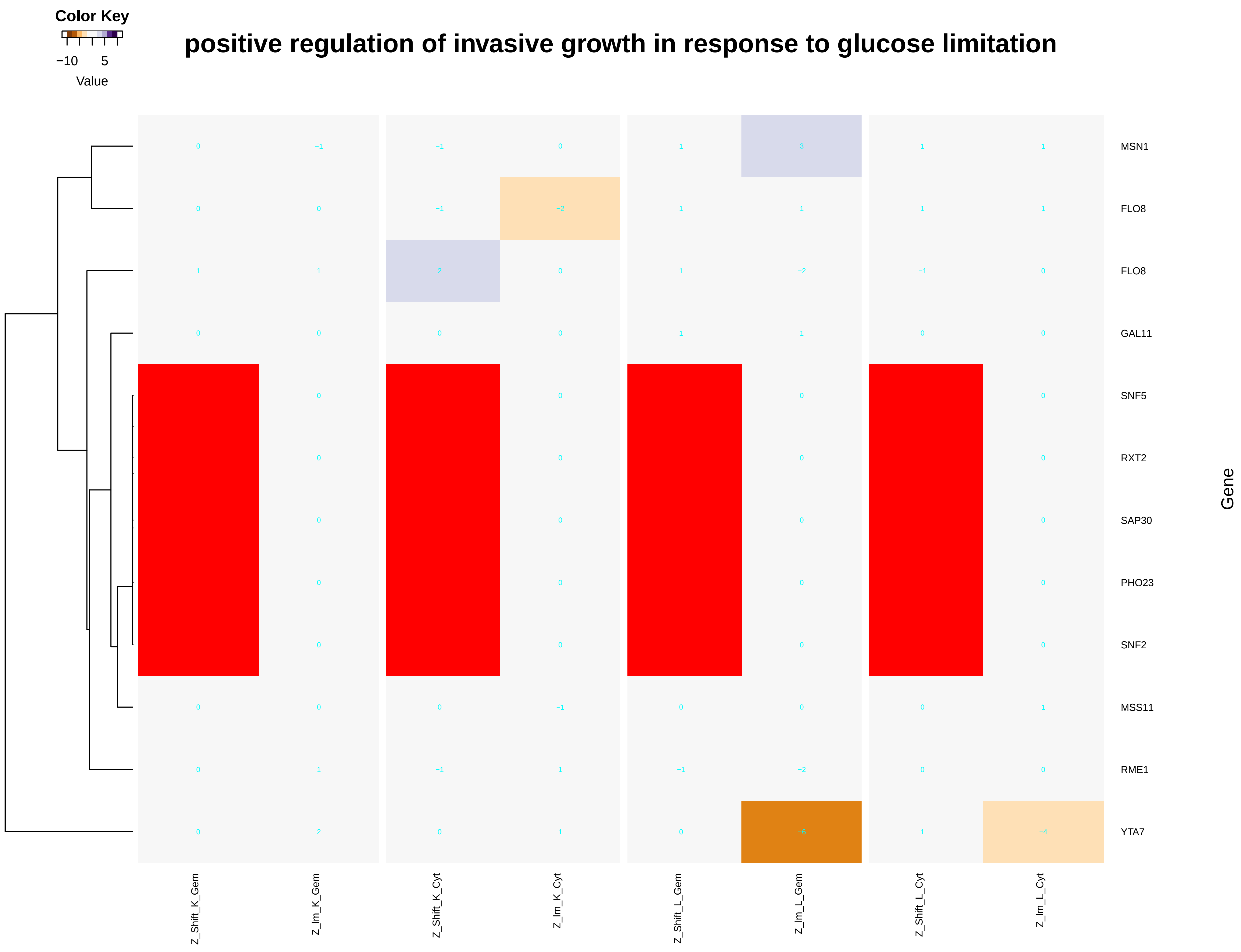

Color Key
−10 5
Value
positive regulation of invasive growth in response to glucose limitation
MSN1
FLO8
FLO8
GAL11
SNF5
RXT2
SAP30
PHO23
SNF2
MSS11
RME1
YTA7
Gene
Z_Shift_K_Gem
Z_Im_K_Gem
Z_Shift_K_Cyt
Z_Im_K_Cyt
Z_Shift_L_Gem
Z_Im_L_Gem
Z_Shift_L_Cyt
Z_Im_L_Cyt

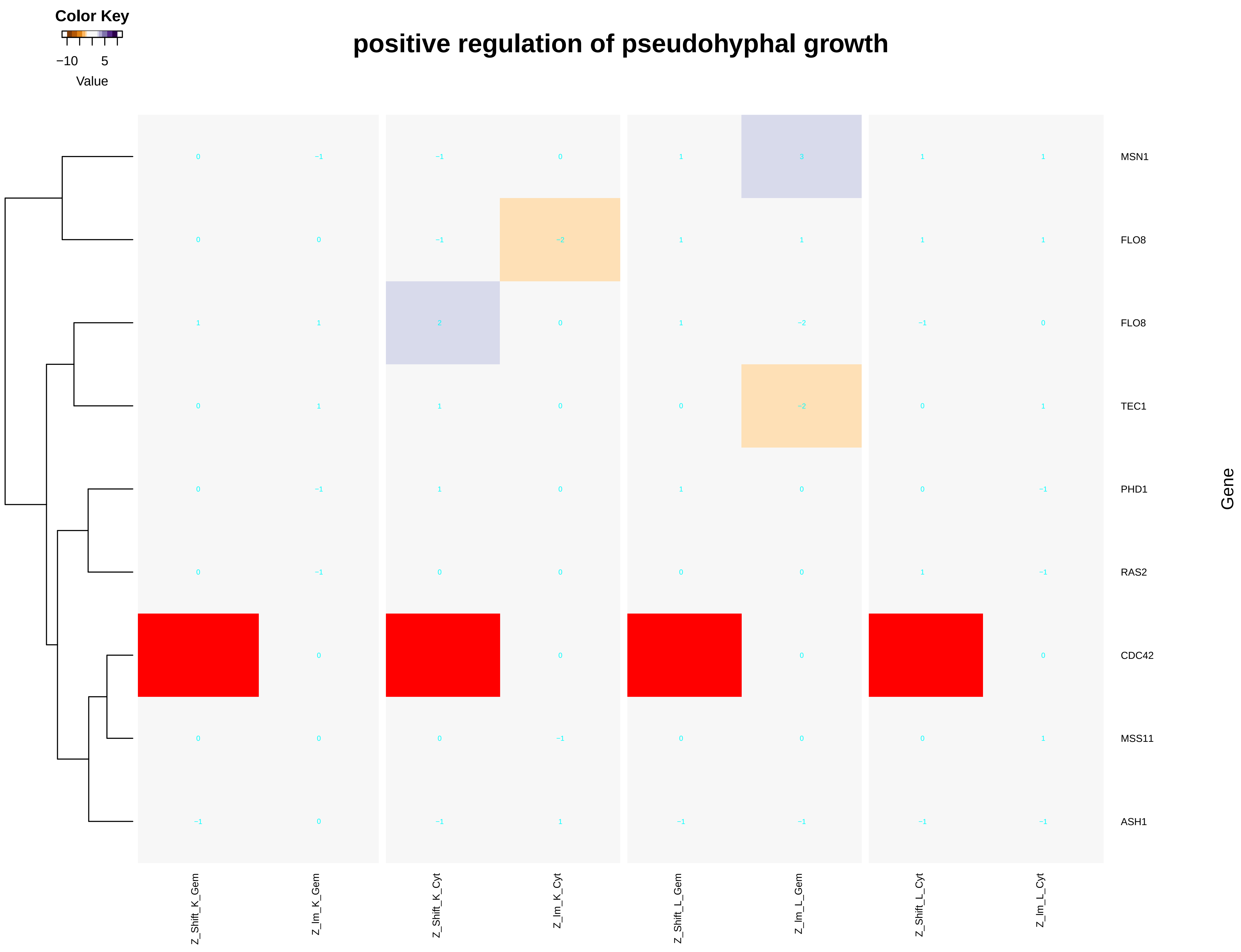

Color Key
−10
5
Value
positive regulation of pseudohyphal growth
MSN1
FLO8
FLO8
TEC1
PHD1
RAS2
CDC42
MSS11
ASH1
Gene
Z_Shift_K_Gem
Z_Im_K_Gem
Z_Shift_K_Cyt
Z_Im_K_Cyt
Z_Shift_L_Gem
Z_Im_L_Gem
Z_Shift_L_Cyt
Z_Im_L_Cyt

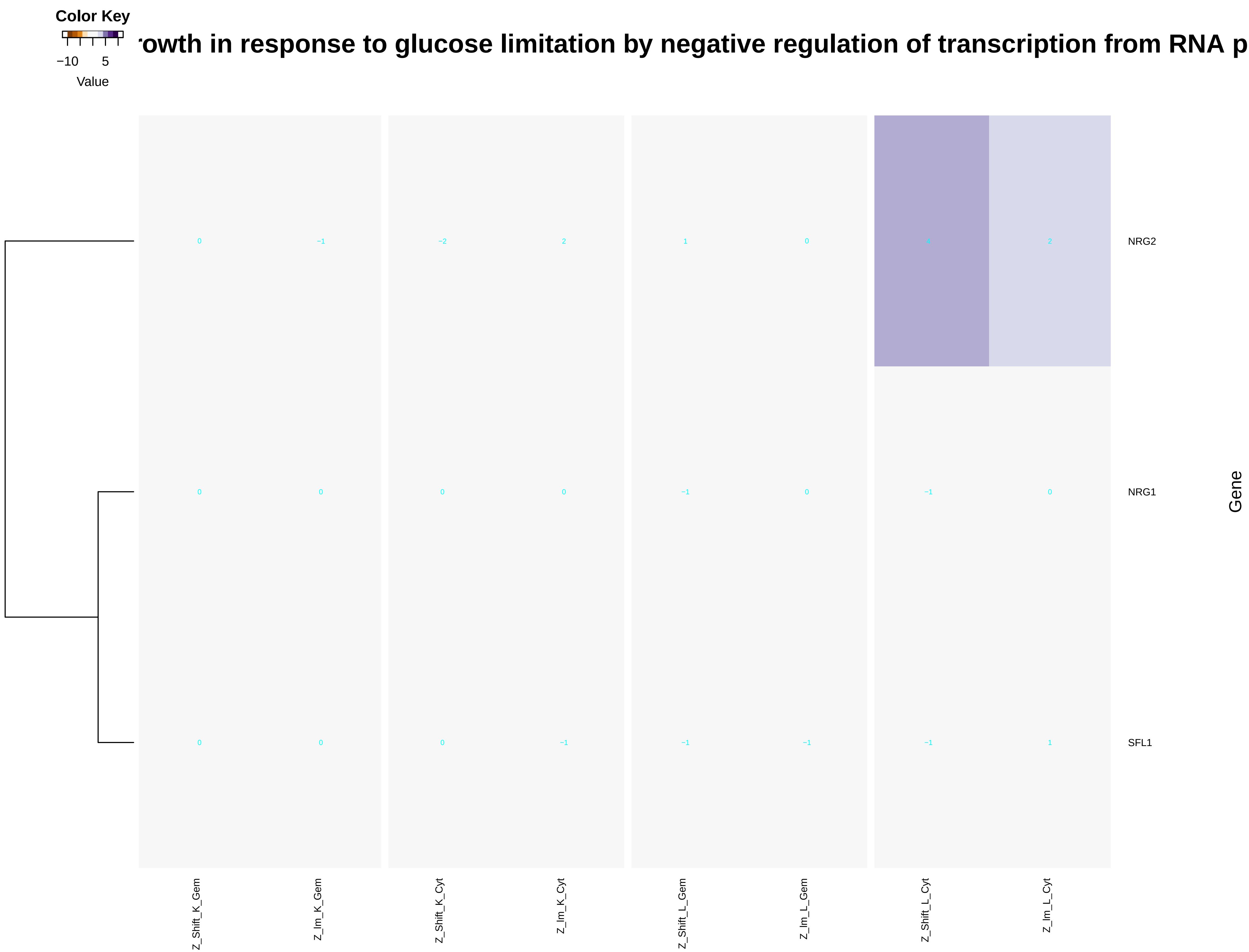

Color Key
−10 5
Value
rowth in response to glucose limitation by negative regulation of transcription from RNA p
NRG2
NRG1
SFL1
Gene
Z_Shift_K_Gem
Z_Im_K_Gem
Z_Shift_K_Cyt
Z_Im_K_Cyt
Z_Shift_L_Gem
Z_Im_L_Gem
Z_Shift_L_Cyt
Z_Im_L_Cyt

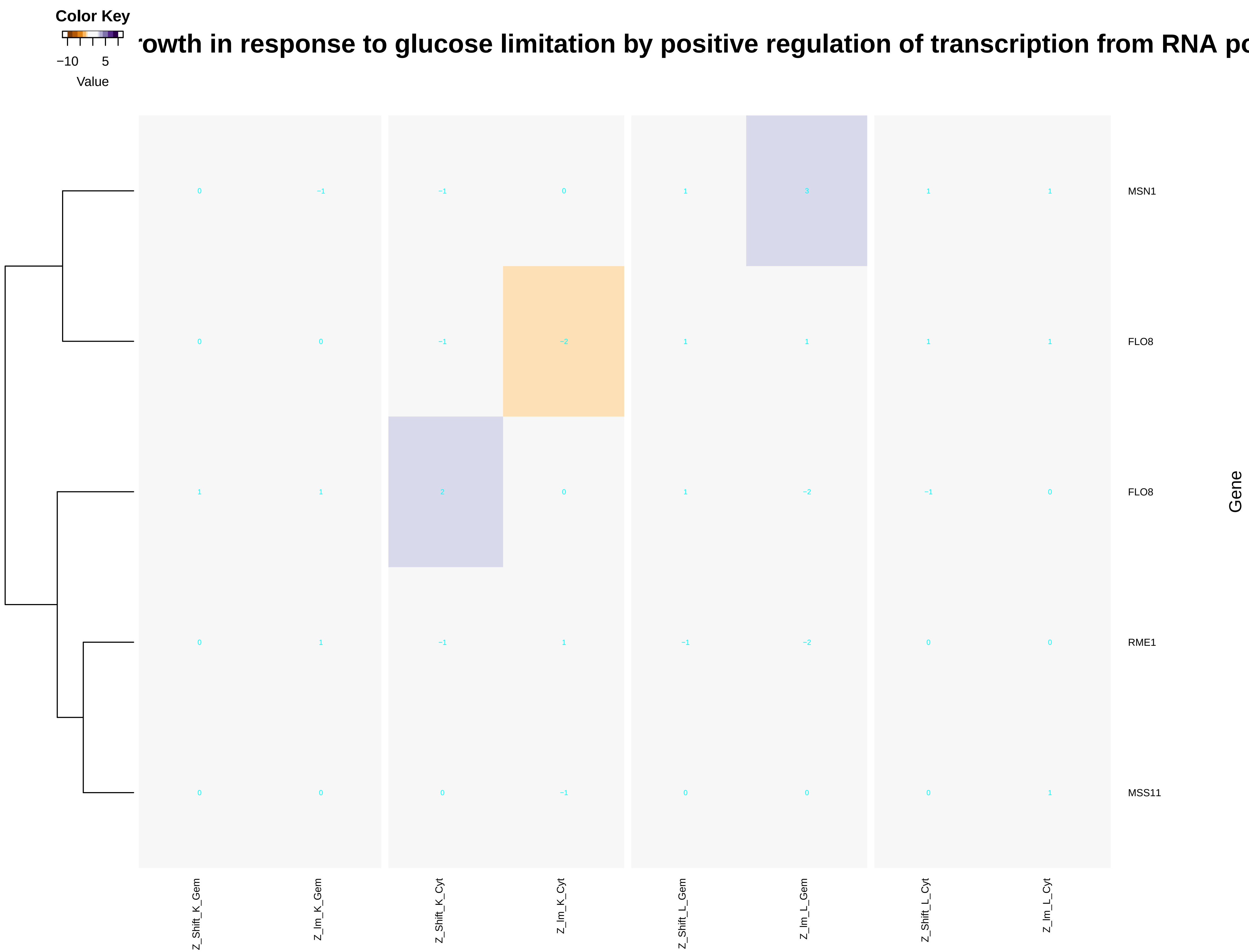

Color Key
−10
5
Value
rowth in response to glucose limitation by positive regulation of transcription from RNA po
MSN1
FLO8
FLO8
RME1
MSS11
Gene
Z_Shift_K_Gem
Z_lm_K_Gem
Z_Shift_K_Cyt
Z_lm_K_Cyt
Z_Shift_L_Gem
Z_lm_L_Gem
Z_Shift_L_Cyt
Z_lm_L_Cyt

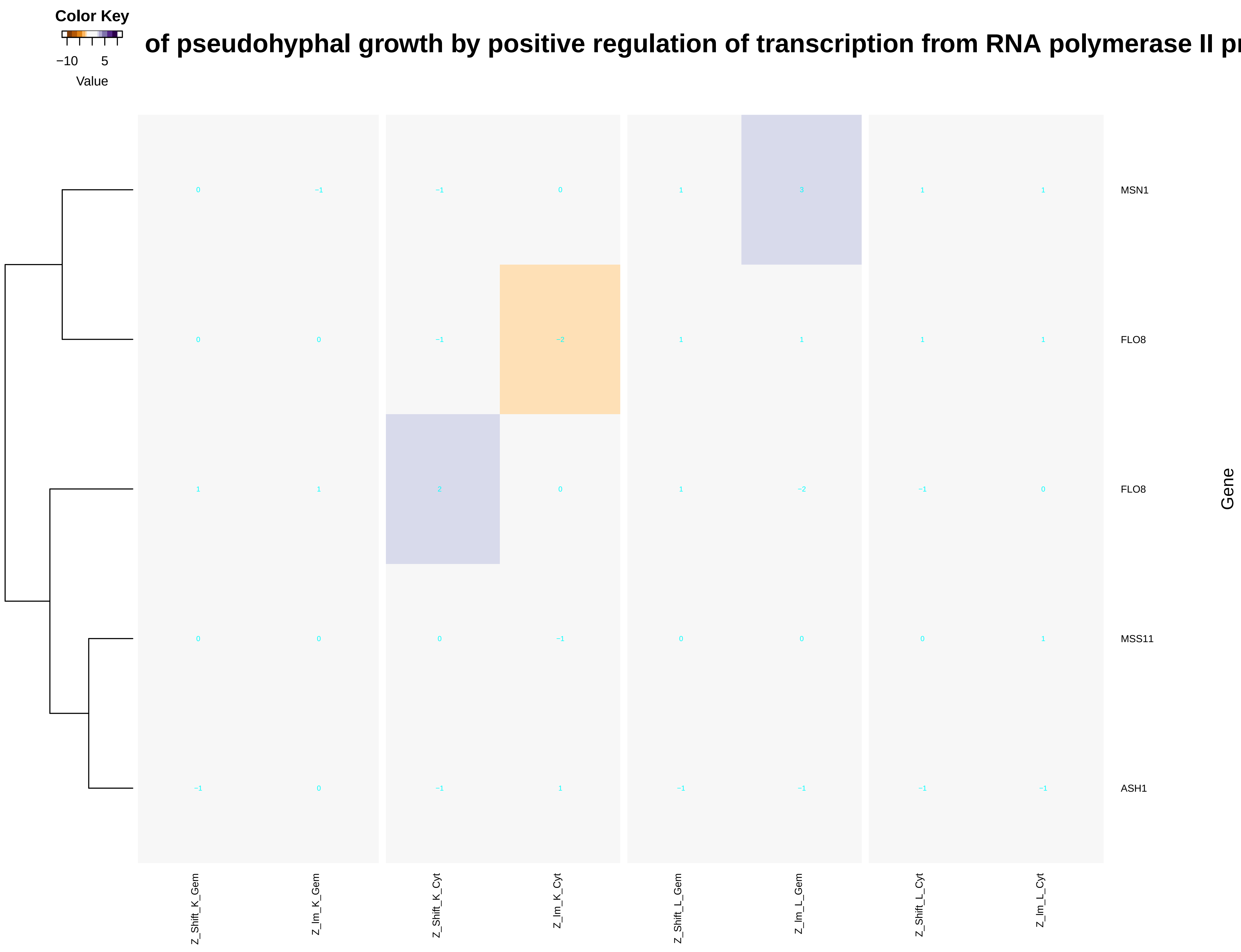
Color Key
−10
5
Value
of pseudohyphal growth by positive regulation of transcription from RNA polymerase II pr
MSN1
FLO8
FLO8
MSS11
ASH1
Gene
Z_Shift_K_Gem
Z_Im_K_Gem
Z_Shift_K_Cyt
Z_Im_K_Cyt
Z_Shift_L_Gem
Z_Im_L_Gem
Z_Shift_L_Cyt
Z_Im_L_Cyt
0
−1
−1
0
1
3
1
1
0
0
−1
−2
1
1
1
1
1
1
2
0
1
−2
−1
0
0
0
0
−1
0
0
0
1
−1
0
−1
1
−1
−1
−1
−1
